# Evolution of Severe Closed Head Injury: Assessing Ventricular Volume and Behavioral Measures at 30 and 90 Days Post-Injury

**DOI:** 10.3390/jcm13030874

**Published:** 2024-02-02

**Authors:** Serena Campana, Luca Cecchetti, Martina Venturi, Francesco Buemi, Cristina Foti, Antonio Cerasa, Carmelo Mario Vicario, Maria Chiara Carboncini, Francesco Tomaiuolo

**Affiliations:** 1 Neurorehabilitation Unit, Auxilium Vitae Volterra, Via Borgo San Lazzero 5, 56048 Volterra, Italy; s.campana@riabilitazione-volterra.it; 2 Social and Affective Neuroscience (SANe) Group, MoMiLab, IMT School for Advanced Studies Lucca, 55100 Lucca, Italy; 3 Department of Translational Research and New Technologies in Medicine and Surgery, University of Pisa, 56126 Pisa, Italy; martina.venturi@uslnordovest.toscana.it; 4 Department of Diagnostic and Interventional Radiology, Azienda Ospedaliera Papardo, 98158 Messina, Italy; francescobuemi@aopapardo.it; 5 Department of Clinical and Experimental Medicine, University of Messina, 98122 Messina, Italy; cristina.foti@unime.it; 6 Institute for Biomedical Research and Innovation (IRIB), National Research Council of Italy, 98164 Messina, Italy; antonio.cerasa@irib.cnr.it; 7 S. Anna Institute, 88900 Crotone, Italy; 8 Pharmacotechnology Documentation and Transfer Unit, Preclinical and Translational Pharmacology, Department of Pharmacy, Health Science and Nutrition, University of Calabria, 87036 Rende, Italy; 9 Department of Cognitive Sciences, Psychology, Education and Cultural Studies, University of Messina, 98125 Messina, Italy; carmelomario.vicario@unime.it

**Keywords:** severe traumatic brain injury, behavioral sequelae, brain imaging, ventricles, rehabilitation

## Abstract

**Background:** Assessing functional outcomes in Severe Closed Head Injury (SCHI) is complex due to brain parenchymal changes. This study examines the Ventricles to Intracranial Volume Ratio (VBR) as a metric for these changes and its correlation with behavioral scales. **Methods:** Thirty-one SCHI patients were included. VBR was derived from CT scans at 3, 30, and 90 days post-injury and compared with Levels of Cognitive Functioning (LCF), Disability Rating Scale (DRS), and Early Rehabilitation Barthel Index (ERBI) assessments at 30 and 90 days. **Results:** Ten patients were excluded post-decompressive craniectomy or ventriculoperitoneal shunt. Findings indicated a VBR decrease at 3 days, suggesting acute phase compression, followed by an increase from 30 to 90 days, indicative of post-acute brain atrophy. VBR correlated positively with the Marshall score in the initial 72 h, positioning it as an early indicator of subsequent brain atrophy. Nevertheless, in contrast to the Marshall score, VBR had stronger associations with DRS and ERBI at 90 days. **Conclusions:** VBR, alongside behavioral assessments, presents a robust framework for evaluating SCHI progression. It supports early functional outcome correlations informing therapeutic approaches. VBR’s reliability underscores its utility in neurorehabilitation for ongoing SCHI assessment and aiding clinical decisions.

## 1. Introduction

Acquired brain injuries stemming from head trauma constitute a complex medical issue, particularly due to the diverse functional outcomes observed among affected individuals [1]. Such variability underscores the intricacies of patient-specific responses to treatment. Furthermore, the prevalence of traumatic brain injuries within the economically active segment of the population underscores its considerable socioeconomic impact, warranting a comprehensive analytical approach [2]. In a recent meta-analysis by Peeters et al. [3], the incidence of traumatic brain injury is notable at 262 per 100,000 people annually. In the United States, a significant proportion of those who survive and are hospitalized—43.3%—are likely to endure long-term disabilities [4,5]. These data underline the critical need for targeted intervention strategies and robust support systems to mitigate the long-term effects on individuals and society. Previous efforts to estimate the evolution of traumatic brain injury have focused on either quantifying the benefits of sequential analysis in real-life situations during large trials [6] or creating prognostic models for cognitive sequelae using Magnetic Resonance Imaging (e.g., [7,8,9,10,11,12,13]). Accurate prognostication from readily available computed tomography (CT) scans would be highly beneficial, particularly in Severe Closed Head Injury (SCHI) patients.

In terms of SCHI-related brain evolution, research has shown that ventricular volume exhibits a slight decrease on the day of the injury scan, followed by a significant increase according to the scan conducted one year after the injury [14,15,16]. The Ventricles to Intra-cranial Volume Ratio (VBR), measured in CT scans, is considered a reliable measure that can be used in traumatic brain injury patients since ventricular borders are sharply defined. Moreover, CT scans are the most utilized and routinely obtained brain images for monitoring the progression of brain changes in traumatic patients together with clinical evolution measures, such as Levels of Cognitive Functioning (LCF), Disability Rating Scale (DRS), and Early Rehabilitation Barthel Index (ERBI).

The objective of this study was to delve into the dynamics of VBR fluctuations as seen in CT scans taken at 3, 30, and 90 days after a SCHI. These intervals were strategically selected to trace the injury’s evolution while mitigating the confounding influences of initial acute care interventions. We plan to establish correlations between VBR changes and the standardized clinical scales, which are integral to neuro-rehabilitation evaluations at the 30- and 90-day marks. CT scans and these behavioral scales are a staple in clinical settings, offering insights without incurring extra costs. By aiming to forecast the functional outcomes for SCHI patients at these early junctures, we hope to provide insights that could shape their ongoing medical treatment and inform the decision-making process regarding their rehabilitation strategies.

## 2. Materials and Methods

### 2.1. Patients Enrollment

The present retrospective observational study was performed on archived data of 31 consecutive severe SCHI patients (20 males, 11 females; mean (SD) age 56 (18); range 25–80 years). All of the patients were of Caucasian ethnicity and were admitted consecutively to the departmental section of “Gravi Cerebrolesioni Acquisite (GCLA)” of the “Azienda Ospedaliero Universitaria Pisana (AOUP)”. All patients were transferred directly from Intensive Care Units and were unconscious at admission to the Intensive Rehabilitation Unit (IRU) (Glasgow Coma Scale [GCS] score of 8 and Level of Cognitive Functioning [LCF] score of 2) with spontaneous breathing without the need for mechanical or physical breathing support other than oxygen therapy. We only included in this study patients with no history of thoracic trauma, neurological or psychiatric disorders, or drug abuse. Our study included patients with SCHI without prior significant medical interventions such as decompressive craniectomy or ventriculoperitoneal shunt procedures that could influence VBR. Any patients displaying signs of post-traumatic hydrocephalus were also to be excluded. Following these criteria, 21 SCHI patients (Table 1 for details) were finally enrolled in this study and underwent clinical and CT examinations at different timepoints. Ten patients were excluded from the analysis: seven of them had undergone decompressive craniectomy due to refractory increased intracranial pressure, and three had received ventriculoperitoneal shunt procedures. None of the included patients developed post-traumatic hydrocephalus during our observation period. 

The present study was conducted after approval of the Local Ethics Committee CEVANO, AUOP Pisa, ID: 12569 regional CINECA or n. 1393/2017 and received approval on 30 March 2017. The surrogate decision-makers of the patients enrolled in the study provided their written informed consent. The original forms were collected and stored at each participant center. All experimental procedures were conducted according to the policies and ethical principles of the Declaration of Helsinki. Data supporting the findings of this study are included in this published article. 

### 2.2. CT Brain Images

**Figure 1:**
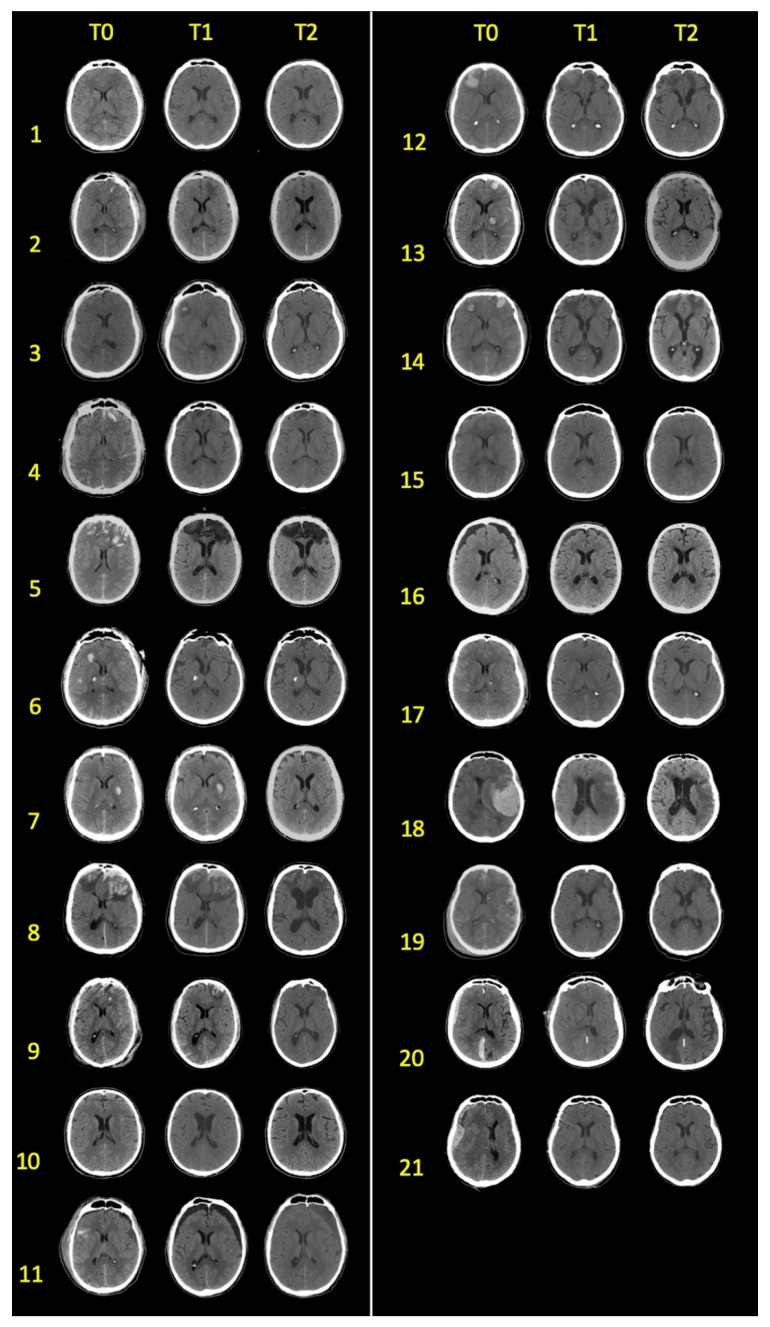
Axial slices of CT scans of each patient collected at 3 (T0), 30 (T1) and 90 (T2) days from injury.

The CT scans were acquired using a GE LightSpeed VCT system with a voxel resolution of 0.488 mm × 0.488 mm (512 × 512 voxel matrix). Axial slices (44 to 64) were obtained, with slice thickness ranging from 2 mm to 5 mm. Every patient underwent three CT brain scans at intervals of 3 (T=), 30 (T1), and 90 (T2) days following the brain injury (Figure 1).

### 2.3. Clinical-Behavioral Measures

Clinical-behavioral assessments for each patient were conducted at 30 and 90 days following the brain injury. The assessment utilized the following scales: Levels of Cognitive Functioning (LCF) [17,18], Disability Rating Scale (DRS) [19], and Early Rehabilitation Barthel Index (ERBI) [20,21]. LCF is employed to describe the spectrum of cognitive impairment levels observed in traumatic brain injury and the subsequent stages of recovery. This scale encompasses eight ascending levels: no response, generalized response, localized response, confused-agitated, confused inappropriate non-agitated, confused-appropriate, automatic-appropriate, and purposeful-appropriate. DRS evaluates the acquired disability of patients with severe head trauma, allowing the monitoring of rehabilitative progress from the coma stage (highest score) through different levels of consciousness and functioning, culminating in a return to regular life (lowest score). This scale comprises eight items, categorized into four sections: arousal and awareness, cognitive capability for self-care functions, physical dependence on others, and psychosocial adaptability for work, household tasks, or school-related activities. Finally, ERBI is an extended version of the Barthel Index, designed to assess performance in activities of daily living [22]. It consists of two sections: negative and positive scores that constitute the essential elements of the original Barthel Index. 

### 2.4. Brain Damage Evaluation

Qualitative measures: Neural damage was assessed using the Marshall scale [23]. The Marshall scale has six categories (1 to 6) that reflect increasing severity based on findings from CT scans. Higher categories indicate poorer prognosis and survival outlook. This scale utilizes factors such as the status of the mesencephalic cisterns, the extent of midline shift in millimeters, and the presence or absence of one or more surgical masses in acute traumatic brain injury [23]. The Marshall CT classification score was used as a classification index of the severity of acute brain injury and should exclusively be assessed within the initial 72-h timeframe.

Quantitative measures: Accurate delineation of the region of interest (ROI) within a CT scan presents challenges, not only in the initial stages of a brain injury but also as the condition evolves. The reliable identification of the boundaries delineating damaged brain tissue from unaffected areas and edematous regions is often obscured, making clear demarcation a complex task. Given this difficulty, our strategy has shifted towards the quantitative evaluation of the brain’s ventricles, structures that remain consistently identifiable on CT scans. The semi-automatic segmentation process we have refined allows us to monitor the ventricular volume, which serves as an indirect marker for the brain’s status. Ventricle enlargement is a recognized indicator associated with more severe brain injuries and is considered predictive of worse outcomes [24].

Measurement of ventricular and intracranial volume: For each participant and scan, we computed the intracranial (ICV) and ventricular volumes (VV), including the lateral ventricles and the third ventricle, using a semi-automatic pipeline adapted from Muschelli and colleagues [25]. The pipeline was implemented in FSL [26] and AFNI [27]. Firstly, we applied a threshold (FSL fslmaths) to CT images, preserving only voxels in the range of 0–100 Hounsfield units. Next, the images were spatially smoothed (AFNI 3dmerge) with a Gaussian kernel of 1 mm^3^ (Full-Width at Half Maximum; FWHM) and brain-extracted (FSL bet; [28]) using the robust brain center estimation (-R option) and a fractional intensity threshold f = 0.01. The ICV was then determined by calculating the number of non-zero voxels present within the brain mask, which was then multiplied by the voxel resolution as quantified by the FSL fslstats tool (https://fsl.fmrib.ox.ac.uk/fsl/fslwiki/Fslutils (accessed on 7 September 2023)). The brain masks, once manually corrected, were applied back to the thresholded CT images via FSL fslmaths. Finally, these brain-extracted images were aligned to the standard MNI152 template using the FSL flirt tool, which facilitated the spatial transformation through affine registration and optimized the process using the correlation ratio as a cost function.

**Figure 2:**
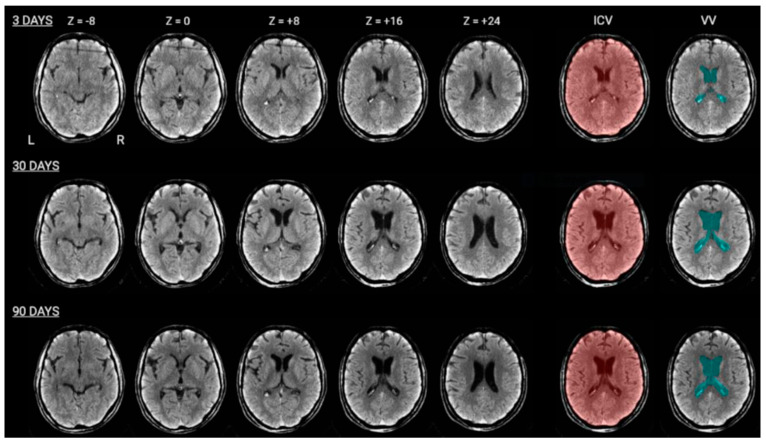
CT scans of the same patient obtained at 3 days (first line), 30 days (second line) and 90 days (third line) after injury. The Z values refer to the coordinate of the horizontal sections in the MNI brain space. The ICV column shows the automatically colored selection of the intracranial volume. The VV columns show the ventricle volume collected automatically and controlled by visual inspection.

To enhance the reliability of the transformation to standard space, any noticeable brain lesions were manually segmented by one of the authors (L.C.) and excluded from the calculation. Next, we computed the inverse transformation from standard space and projected back to the original space a mask of the ventricular system (MNI152_T1_2mm_VentricleMask.nii.gz provided with the FSL software). Voxels with intensities in the range of 0–15 Hounsfield units, located within this mask, constituted the segmentation mask for the participant’s ventricles (Figure 2). The two raters, S.C. and F.T., who were blind to the study participants or the CT scan sessions, reviewed and corrected the brain extraction masks visually. The raters employed the Display software, a resource developed by J.D. MacDonald at the Brain Imaging Centre of the Montreal Neurological Institute, (http://www.bic.mni.mcgill.ca/ServicesSoftware/ServicesSoftwareMincToolKit (accessed on 17 December 2023)). The software supports voxel labeling on individual CT scan slices, simultaneously presenting an integrated view of the sagittal, axial, and coronal planes. With the ‘mouse brush’ tool in Display, raters could, when necessary, enhance the delineation of voxel regions for the brain extraction masks, using three-dimensional visualization techniques to improve the precision of the masks’ automatic selections (Figure 2). The VV (cm^3^) was determined by multiplying the number of voxels within the final mask by the voxel resolution (FSL fslstats).

Ventricles to Intra-cranial Volume Ratio (VBR): Ventricular volume normalization was corrected for variations in head size, dividing the VV by the ICV and multiplying by 1000 (VV/ICV × 1000; [14,29]). This ratio was used to adjust for differences in brain size among participants.

All tools and commands needed to replicate the current method are listed in this section, and a template script can be accessed through this link: https://osf.io/ep38x/ (accessed on 17 December 2023). The CT scan data are stored on a specific hard-disk and are available from the corresponding authors upon reasonable request, respecting privacy regulations.

### 2.5. Data Analysis

Data analysis was performed using Jamovi—open statistical software (https://www.jamovi.org/ (accessed on 27 February 2023)). We conducted a mixed within- and between-factor ANOVA to analyze the VBR: the within-factor was VBR across the three sampling times (3 days vs. 30 days vs. 90 days; Table 2 for details), while the between-factor was the group (young patients vs. old patients). The between-factor group was used to assess the potential impact of age groups. Post hoc testing was carried out using the Bonferroni test. All statistical analyses had a two-tailed α level of <0.05 for defining significance. Spearman–Brown rank correlation was used across the three sampling times of anatomical sampling of the VBR and the Marshall scores. Spearman–Brown rank correlation was also used across the three timepoints of anatomical sampling to correlate VBR and Marshall scores to the behavioral measures represented by the LCF, GOS, DRS and ERBI scores collected at 30 and 90 days after the injury.

## 3. Results

**Figure 3:**
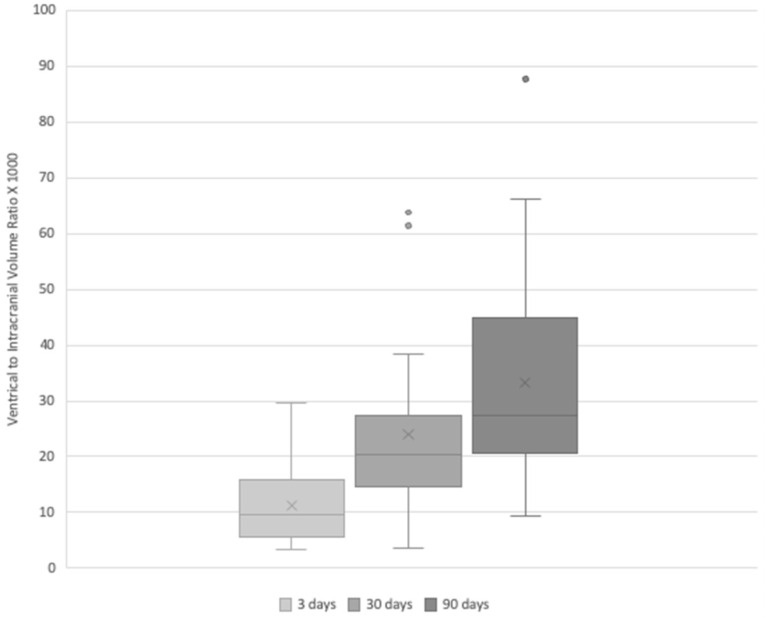
Ventricles to Intracranial Volume Ratio (VBR) measured at 3, 30, and 90 days post-injury in patients with severe closed head injury. Each box represents the interquartile range of the data, with the horizontal line indicating the median, and the ‘X’ marking the mean. Outliers are represented by dots. The data points for each period are overlaid on the box plots to show individual variations. The increasing trend in VBR over time highlights the brain atrophy progression post-injury. Each comparison was significantly different.

Twenty-one SCHI patients were included in the analysis. Behavioral analysis showed a significant clinical improvement over time (See Table 1). Significant improvements after 90 days were detected for LCF (t = 12.6; *p*-value < 0.001); DRS (t = 13.1; *p*-level < 0.001) and ERBI (t = −17.2; *p*-level < 0.001). No significant difference was found between young patients and old patients (F = 1.891, n.s.). Post hoc comparisons with Bonferroni correction (*p* = [0.05/3], 0.016) confirmed these VBR increases, specifically between 3 days and 30 days (t = −4.012, *p* < 0.001), between 30 days and 90 days (t = −2.863, *p* = 0.020), and between 3 days and 90 days (t = −6.875, *p* < 0.001; Figure 3).

Regarding the VBR volumes, there was a significant increase in VBR values from 3 days to 90 days (F = 23.853; *p* < 0.001; mean ± sd: after 3 days: 10 ± 6, after 30 days: 19 ± 9, after 90 days: 26 ± 14).

The Marshall score was positively correlated only with the VBR collected at 90 days (r = 0.565; *p* = 0.008) after the injury. The Marshall score did not show significant correlations with any of the behavioral scales (LCF, DRS, ERBI) administered at 30 and 90 days after the trauma.

Correlations between the VBR measured at 3 and 30 days and the behavioral scales (LCF, DRS, ERBI) administered at 30 and 90 days after the trauma were not found to be significant.

Significant correlations with the VBR measured at 90 days were found for the DRS and ERBI collected at 90 days after the injury. It should be noted that the VBR at 90 days was negatively correlated with ERBI scores at 90 days (r = −0.509; *p* = 0.018), while significant positive correlations between the VBR measured at 90 days and the DRS were observed (r = 0.496; *p* = 0.022; Table 3).

## 4. Discussion

We examined the evolution of SCHI in patients using the VBR, an indirect measure of the neural state of the brain that can be reliably detected from routine CT scans. In comparison to previous research, our study offers two significant methodological innovations: (a) the employment of an anatomical metric that makes it simple and automatic to detect the signal of cerebral spinal fluid; (b) the longitudinal evaluation of brain atrophy progression at 3, 30, and 90 days post-injury to assess the modification of acute-subacute effects on ventricular volume.

Several studies have investigated atrophic brain rates after traumatic brain injury in a time frame of typically less than 2 months [30,31,32,33,34,35,36,37]. However, these analyses may be influenced by acute and subacute effects of injury, such as edema or hemorrhage, which can be difficult to discern between pathologic and healthy tissue, especially when there is edema present and the signals from white and gray matter can appear identical. Moreover, unlike other studies (e.g., [30,31,32]), which included patients with severe and moderate head trauma, we evaluated only patients who experienced SCHI without decompressive craniectomy for increased intracranial pressure refractory to medical management.

In SCHI patients, ventricular volume variation reflects the status of the brain parenchyma [31,38]. A reduction in VBR indicates brain damage resulting from edema and/or hemorrhage [31,38]. During the sub-acute phase, there is an increase in VBR, attributed to the resolution of edema and/or hemorrhage and potentially the development of ex-vacuo hydrocephalus due to neural loss in brain parenchyma [31,38,39]. Edema resolution significantly depends on the severity of traumatic brain injury and the therapies administered (e.g., mannitol, craniectomy; [40,41]). Following the reduction of edema and/or hemorrhage, the ventricles may re-expand, leading to an increase in their volume. Typically, a decrease in total brain volume begins approximately 3 weeks after moderate to severe traumatic brain injury and continues for 8–12 months [38,42]. Thus, at 3 days after trauma, acute injuries such as hemorrhage or edema are observed, whereas at 30 and 90 days, the brain undergoes global or local atrophic changes. The increase in ventricular size above the pre-injury state occurs because of the space left by the atrophy caused by damaged brain tissue, with larger ventricles indicating more severe atrophy [31].

We observed a gradual enlargement of the ventricles in the examined patients, driven by two phenomena: acute phase compression due to edema and/or hemorrhage and post-acute phase enlargement due to brain atrophy. The VBR exhibited a positive correlation with the traditional Marshall score, known to be an index of brain damage severity and its behavioral evolution [43]. Specifically, we found that higher Marshall scores were associated with larger ventricular dimensions at 90 days after injury, indicating that the Marshall score can estimate brain atrophy as early as 90 days post-trauma. The prognostic power of the Marshall score in classifying cerebral damage is well-established [44,45], making it the most used classification system in clinical practice [43].

Notably, the Marshall score did not correlate with any of the behavioral scales used (LCF, DRS, and ERBI) at the 30- and 90-day sampling points. The lower sensitivity of the Marshall score was likely a result of the limited number of groups in which the patients were divided in this study [43]. On the other hand, the VBR allowed us to categorize patients across a continuous broad range of values, leading to better sensitivity. Furthermore, while the literature suggests that the Marshall score can only be calculated using neuroimaging within the first 72 h from trauma [23], we demonstrated that the analysis of VBR collected at 90 days from injury was correlated at 90 days after injury.

### Limitations

One limitation of this study is the semi-automatic measurement of ventricular volumes, which requires visual inspection and potential corrections of computational selections of ROI. Specifically, due to variability in choroid plexus density, some portions may remain undetected computationally, necessitating manual selection to include them in the overall ventricle volume calculation. Another limitation of the study is the wide age range included in the sample. Age might be a confounding factor in the evolution of the clinical state. To account for this confounding factor, we performed an ANOVA analysis with VBR across three sampling times as the within-factor and patient age group as the between-factor. This approach allowed us to statistically consider age by directly comparing younger and older patient groups. Furthermore, we included age as a covariate in our statistical model to control for its effects on VBR. We believe this method adequately addresses the concern raised and provides a reliable analysis of VBR changes that are not solely attributable to natural neurodegenerative processes. However, further investigations are needed to understand its specific impact.

The study’s limitations, including the semi-automatic measurement of ventricular volumes, call for further research to refine the methods and assess the potential impact of age on clinical outcomes. Nevertheless, the ability to estimate brain atrophy and its correlations with functional outcomes could be highly beneficial for the early estimation of functional outcomes in SCHI patients, aiding in their illness management and guiding appropriate therapeutic interventions. The use of VBR as a reliable and easily accessible measure from routine CT scans offers a valuable tool for neuro-rehabilitation units to track brain evolution in SCHI patients. Integrating VBR with traditional behavioral assessments provides a comprehensive evaluation of SCHI progression, enabling clinicians to make more informed decisions about patient care and therapy planning. Overall, our study contributes valuable insights into the evolving field of traumatic brain injury research and encourages further investigation into the clinical utility of VBR as a prognostic indicator in the management of these patients.

Finally, given that this study was observational, we acknowledge that we could not eliminate the effects of different treatments. However, our analyses focused on the evolution of VBR over time, and the significant findings suggest a pattern that is less likely to be solely due to varied treatment approaches. We suggest that future prospective studies could be designed to control and directly compare the effects of different treatment modalities on VBR.

## 5. Conclusions

In this study, we explored the trajectory of SCHI by employing the VBR as a surrogate marker for parenchymal volume changes, as evidenced by routine CT scans. Our analysis delineated a significant increment in VBR from 3 to 30 to 90 days post-injury. This rise encapsulates the acute phase of brain compression, potentially due to edema and hemorrhage, and the subsequent phase indicative of brain atrophy. Notably, VBR’s positive correlation with the established Marshall score, a conventional metric for quantifying brain injury severity, underscores its prospective utility as an early indicator of brain atrophy, particularly 90 days following the brain injury.

In contrast, the Marshall score’s weak correlation with behavioral scales such as the LCF, DRS and ERBI across the same intervals accentuates VBR’s superior sensitivity in capturing the continuous variations in brain status, potentially offering a reduction in measurement variability. Our study supports the importance of continued research to refine prognostic instruments, advocating for the adoption of more personalized clinical methods that can seamlessly become part of standard care practices.

Looking ahead, the fusion of advanced neuroimaging technologies with innovative machine learning techniques, like convolutional neural networks e.g., [46], could be a game-changer for SCHI research. This synergy promises to enhance our capabilities in processing and interpreting brain imaging and behavioral data, thereby deepening our grasp of SCHI’s underlying pathophysiological complexities. These technological advances herald the advent of more personalized and impactful therapeutic interventions, enabling a more precise prediction of patient outcomes and informing the development of rehabilitation plans and neuroprotective interventions.

Supported by advancements in data-driven precision medicine, physicians will more effectively tailor treatment strategies in the ongoing development of treatment paradigms, improving the quality of life and the recovery paths for those affected by SCHI. This enhancement in clinical effectiveness, along with a stronger emphasis on patient-centered care, provides an optimistic perspective for the future management of SCHI, in step with the expansive goals of therapy.

## Figures and Tables

**Table 1:** Behavioral data of each patient collected at 30 and 90 days from injury.

			30 Days			90 Days		
Patient	Sex	Age	LCF	DRS	ERBI	LCF	DRS	ERBI
1	m	25	4	18	−125	6	2	100
2	m	40	4	18	−95	8	4	65
3	m	41	4	20	−110	7	3	90
4	m	42	4	15	0	6	4	100
5	m	43	5	23	−150	8	3	95
6	m	49	3	23	−225	5	8	45
7	m	51	2	26	−225	6	12	55
8	m	52	4	21	−225	5	14	0
9	m	53	4	12	−55	8	3	95
10	m	54	3	26	−225	6	14	55
11	m	62	4	17	−50	6	3	80
12	m	66	5	16	−150	8	3	65
13	m	68	3	24	−225	5	12	0
14	m	74	5	18	−50	8	8	80
15	f	25	5	13	0	8	2	100
16	f	61	5	16	−90	7	3	65
17	f	61	2	26	−225	6	10	40
18	f	76	2	26	−275	3	21	0
19	f	79	2	25	−225	3	23	0
20	f	80	4	18	−170	6	19	10
21	f	80	3	15	−170	6	4	55
mean/ [median]		51	[4]	19.5	−130.8	[6]	6.3	66.4
sd/[range]		13.9	[2–5]	4.5	83.07	[5–8]	4.4	31.8

Levels of Cognitive Functioning (LCF), Disability Rating Scale (DRS), and Early Rehabilitation Barthel Index (ERBI). Data are shown as mean or median values.

**Table 2:** Anatomical measures: Marshall score, Intra Cranial Volume (ICV), Ventricles Volume (VV) at 3, 30 and 90 days from injury.

Patient	Marshall Score	ICV (cm^3^)	VV 3 Days (cm^3^)	VV 30 Days (cm^3^)	VV 90 Days (cm^3^)
1	3	1357	5.92	28.50	27.98
2	3	1722	21.98	34.12	34.56
3	4	1658	16.76	16.40	37.80
4	3	1497	5.41	20.94	21.25
5	6	1735	7.31	29.51	35.66
6	3	1437	8.63	35.33	37.44
7	3	1562	6.59	6.63	18.81
8	6	1612	37.05	32.72	94.28
9	5	1715	29.05	30.54	53.47
10	3	1520	12.42	29.44	32.20
11	3	1495	10.99	15.81	14.13
12	6	1608	16.56	43.82	53.10
13	3	1562	25.42	55.68	34.62
14	3	1829	27.06	70.20	76.49
15	2	1339	4.43	19.70	13.11
16	3	1301	12.01	24.10	27.14
17	3	1330	6.51	5.84	19.69
18	6	1359	8.57	35.64	65.00
19	3	1236	7.61	41.27	54.37
20	6	1643	20.15	15.69	68.28
21	4	1164	22.68	23.79	23.71
mean		1508.6	14.9	29.3	40.1
sd		181.2	9.3	15.3	21.9

**Table 3:** Correlation with behavioral data at 30 and 90 days from injury and VBR at 3, 30 and 90 days from injury.

	Timepoints	VBR 3 Days	VBR 30 Days	VBR 90 Days
LCF	30 days	0.076 (0.744)	0.009 (0.970)	−0.049 (0.831)
	90 days	0.033 (0.886)	−0.215 (0.348)	−0.294 (0.196)
DRS	30 days	−0.186 (0.420)	0.050 (0.831)	0.216 (0.347)
	90 days	0.193 (0.402)	0.186 (0.420)	0.496 (0.022) *
ERBI	30 days	−0.076 (0.745)	−0.215 (0.350)	−0.415 (0.061)
	90 days	−0.347 (0.123)	−0.284 (0.212)	−0.509 (0.018) *
MARSHALL	30 days	0.432 (0.051)	0.039 (0.866)	0.565 (0.008) *

Ventricles to Intra-cranial Volume Ratio (VBR), Levels of Cognitive Functioning (LCF), Disability Rating Scale (DRS), and Early Rehabilitation Barthel Index (ERBI). Data were reported as Spearman rho value (*p* value) * *p* < 0.05.

## Data Availability

The behavioral testing is reported in full in Table 1. Upon reasonable request, neuroimaging data and scripts can be made available to interested parties by contacting the corresponding authors. Data associated with the article are stored in a separate specific hard disk.
